# Photoresponsive Surface Molecularly Imprinted Polymers for the Detection of Profenofos in Tomato and Mangosteen

**DOI:** 10.3389/fchem.2020.583036

**Published:** 2020-10-09

**Authors:** Mei-jun Chen, Hai-lin Yang, Ya-min Si, Qian Tang, Cheuk-fai Chow, Cheng-bin Gong

**Affiliations:** ^1^The Key Laboratory of Applied Chemistry of Chongqing Municipality, College of Chemistry and Chemical Engineering, Southwest University, Chongqing, China; ^2^Department of Science and Environmental Studies, The Education University of Hong Kong, Tai Po, Hong Kong

**Keywords:** photoresponsive materials, surface molecularly imprinted polymer, azobenzene, profenofos, organophosphorus pesticide

## Abstract

As a moderately toxic organophosphorus pesticide, profenofos (PFF) is widely used in agricultural practice, resulting in the accumulation of a high amount of PFF in agricultural products and the environment. This will inevitably damage our health. Therefore, it is important to establish a convenient and sensitive method for the detection of PFF. This paper reports a photoresponsive surface-imprinted polymer based on poly(styrene-*co*-methyl acrylic acid) (PS-*co*-PMAA@PSMIPs) for the detection of PFF by using carboxyl-capped polystyrene microspheres (PS-*co*-PMAA), PFF, 4-((4-(methacryloyloxy)phenyl)diazenyl) benzoic acid, and triethanolamine trimethacrylate as the substrate, template, functional monomer, and cross-linker, respectively. PS-*co*-PMAA@PSMIP shows good photoresponsive properties in DMSO/H_2_O (3:1, v/v). Its photoisomerization rate constant exhibits a good linear relationship with PFF concentration in the range of 0~15 μmol/L. PS-*co*-PMAA@PSMIP was applied for the determination of PFF in spiked tomato and mangosteen with good recoveries ranging in 94.4–102.4%.

## Introduction

Profenofos (PFF), [O-(4-bromo-2-chlorophenyl) O-ethyl S-propyl phosphorothioate], is widely used to control pest strains that are resistant to chlorpyrifos and other organophosphorus pesticides in fruit trees, vegetables, and cotton (He et al., 2010; Dadson et al., 2013). However, PFF is moderately to highly toxic to animals with an LD50 of 500 mg/kg (Gotoh et al., 2001; Ma et al., 2019). Thus, it is essential to detect PFF in agricultural products. Common detection methods for PFF include gas chromatography (Yang et al., 2012), high-performance liquid chromatography (HPLC) (Raharjo et al., 2009), biosensors (Li C. et al., 2018; Selvolini et al., 2018; Xiong et al., 2018), and colorimetric and fluorescent sensors (Zhang et al., 2014; Li X. et al., 2018; Kovida and Koner, 2020). Although those methods are sensitive, they are time- and reagent-consuming and require a professional operator, and sample preparation is complex. Furthermore, most of them demand expensive instruments.

Molecularly imprinted polymers (MIPs) are synthesized by copolymerizing functional monomers and cross-linkers in the presence of template molecules. The elution of template molecules makes it possible for the formation of tailor-made recognition cavities complementary to the template molecule in shape, size, and chemical functionality in the highly cross-linked polymer matrix. Meanwhile, those imprinted cavities are endowed with the established ability to recognize the target analyte, i.e., template molecules (Xu et al., 2013). MIPs are widely applied in purification and separation (Li et al., 2015; Gong et al., 2017a; He et al., 2018; Zhang et al., 2018; Gomez-Arribas et al., 2019), chiral recognition (Rutkowska et al., 2018; Zhao et al., 2019; Li et al., 2020), chemo- and bio-sensing (Altintas et al., 2016; Gong et al., 2017b; Ding et al., 2020; Kalecki et al., 2020), and catalysis and degradation (Liu et al., 2015; Zheng et al., 2017; Boitard et al., 2019; Muratsugu et al., 2020) due to its physical stability, thermal stability, low cost, and ease of preparation.

Stimulus-responsive molecularly imprinted polymers are generally prepared by introduction of stimulus-responsive moieties into MIPs. These MIP materials can respond to specific external stimuli such as temperature, light, magnetic field, solvent composition or solvent polarity, pH, or electric field through altering itself greatly at solubility, molecular chain structure, surface structure, and swelling or dissociation behavior. Among them, photoresponsive MIPs (PMIPs) attract great research attention because of the advantages of clean, remote control and no damage to sample (Schumers et al., 2010). PMIPs make it possible for template extraction, recognition, and release under external photo-irradiation at a certain wavelength (Alaei et al., 2018). The photoresponsive azobenzene moiety is generally introduced into MIP/SMIP by using azobenzene derivatives as a functional monomer (Gong et al., 2006, 2016). Good achievements have been obtained in PMIPs in recent years. Azobenzene derivatives containing hydrophilic –SO_3_H and –COOH groups were designed as the functional monomers to realize the photo-response of PMIPs in aqueous solution (Tang et al., 2012, 2016). The *o*-methyl and *o*-methoxy azobenzene derivatives were designed to realize visible-light response for both trans to cis and cis to trans photoisomerization (Liu et al., 2018, 2020; Gong et al., 2019). Photoresponsive SMIPs (PSMIPs) and hollow MIPs were fabricated to solve the problem of template being embedded in traditional PMIPs (Gong et al., 2016, 2017c; Yang et al., 2018). PSMIPs have potential applications in drug delivery, electrochemical sensor, and environmental pollutant detection. However, the application in detecting pesticide residues by PMIPs or PSMIPs has been rarely reported.

In this article, we focused on developing a method to detect trace PFF in agricultural products such as mangosteen and tomato by using a photoresponsive surface molecularly imprinted polymer PS-*co*-PMAA@PSMIP. In the presence of the PFF template, MPABA and TEAMA were copolymerized on the surface of PS-*co*-PMAA by using potassium persulfate (KPS) as the initiator. PS-*co*-PMAA@PSMIPs were obtained after the subsequent removal of the PFF template, and specific recognition sites with memory of the shape, size, and functionality of PFF template were formed within PS-*co*-PMAA@PSMIPs. PS-*co*-PMAA@PSMIPs were applied to detect trace PFF in complex vegetable and fruit sample matrices. PS-*co*-PMAA@PSMIPs have a good binding capacity for PFF, a quick mass transfer rate, and excellent cycling stability on photo-regulated release and uptake of PFF.

## Experiment

### Materials and Instruments

Phenol (≥99.5%), *p*-aminobenzoic acid (99%), methacrylic anhydride (99%), triethylamine (99%), hydrochloric acid, KPS (99.5%), thionyl chloride (SOCl_2_, 99%), methacrylic acid (99%), styrene (≥99%), triethanolamine (98%), PFF (98%), chlorpyrifos (CPF, 95%), chlorpyrifos-methyl (CPFm, 98%), MgSO_4_, NaNO_2_, NaOH, ethanol, N,N-dimethylformamide (DMF), and dimethyl sulfoxide (DMSO) were purchased from Aladdin Co. Ltd. (Shanghai, P. R. China). High-purity hydrogen and high-purity nitrogen were purchased from local gas companies.

^1^H NMR and ^13^C NMR were recorded on a Bruker AV-600 NMR instrument at an ambient temperature (25°C) using tetramethylsilane as an internal standard. Ultraviolet–visible (UV–Vis) spectra were obtained using a UV-4802 spectrophotometer (UNICO (Shanghai) Instruments Co. Ltd., P. R. China). A CEL S-500 Xe light was used as a light source (Beijing Zhong Jiao Jin Yuan Ke Ji Co. Ltd., P. R. China), and wavelengths of 365 and 440 nm were, respectively, selected using 365- and 440-nm filters. The morphologies of silica microspheres, PS-*co*-PMAA@PSMIPs, and photoresponsive surface non-imprinted polymers based on PS-*co*-PMAA (PS-*co*-PMAA@PSNIPs) were identified by scanning electron microscopy (SEM; S-4800, Hitachi, Tokyo, Japan) and transmission electron microscopy (TEM; FEI Talos F200X, USA). Fourier transform infrared spectroscopy (FT-IR) was recorded on a Perkin-Elmer Model GX spectrometer using a KBr pellet method. Nitrogen adsorption–desorption analysis was conducted at 77 K on an Autosorb-1 apparatus (Quantachrome, USA). Specific surface areas and pore diameters were calculated using the Brunauer–Emmett–Teller (BET) and Barrett–Joyner–Halenda (BJH) models, respectively.

### Synthesis of Functional Monomer, PS-*co*-PMAA Substrate, and Cross-Linker

The functional monomer MPABA was synthesized according to the method reported by Gong et al. (2006) by using *p*-aminobenzoic acid as the starting material. The cross-linker TEAMA was synthesized according to the method reported by Li et al. (2015) by using methacrylic acid, thionyl chloride, and triethanolamine as the starting materials. PS-*co*-PMAA substrate was synthesized according to the method reported by Yang et al. (2018) by using styrene and methacrylic acid as the raw materials.

### Synthesis of PS-*co*-PMAA@PSMIPs and PS-*co*-PMAA@PSNIPs

PS-*co*-PMAA (50.00 mg) was dispersed in 10 mL DMSO/H_2_O (3:1, v/v) solution in a 100-mL three-necked bottle, sonicated for 30 min, and then stirred for 2 h to ensure the formation of stable suspension. MPABA (31.0 mg, 0.10 mmol) and PFF (7.5 mg, 0.02 mmol) were dissolved in a mixed solvent of DMSO/H_2_O (10 mL; 3:1, v/v), which was slowly added to the PS-*co*-PMAA suspension. The suspension was stirred for 12 h to ensure a complete interaction between MPABA and PFF through hydrogen bond. Subsequently, ~5 mL TEAMA solution (70.7 mg, 0.20 mmol) in DMSO/H_2_O (3:1, v/v) was added by a syringe. The mixture was stirred for 30 min and evacuated for 10 min, and then nitrogen gas was bubbled below the liquid level of the suspension for 10 min to remove oxygen. During this process, 5 mL KPS solution (14.0 mg KPS was dissolved in 5 mL distilled water) was added. The mixture was placed in an oil bath at 80°C for 24 h in a nitrogen atmosphere. After being cooled to room temperature, the product was separated by centrifuging at 10,000 rpm for 5 min, washed with ethanol and water (1:1, v/v) until the supernatant was colorless, dried at 50°C, and ground to powder. This powder was further Soxhlet-extracted with methanol and acetic acid (4:1, v/v) solution for 48 h and then Soxhlet-extracted with methanol for 24 h to fully elute the PFF. Finally, the eluted powder was dried at 50°C to obtain PS-*co*-PMAA@PSMIPs (123.2 mg). PS-*co*-PMAA@PSNIPs were synthesized using the same way but without the addition of template PFF.

### Spectroscopic Characterization and Photoisomerization Studies

Spectroscopic characterization and photoisomerization of MPABA and PS-*co*-PMAA@PSMIPs were performed in DMSO/H_2_O (3:1, v/v) according to the method reported by Gong et al. (2016). The photoisomerization kinetic rate constants (*k*) from trans to cis and cis to trans are calculated according to Equation 1,

(1) $$\begin{array}{r} \ln\;\frac{A_{0}-A_{\infty }}{A_{t}-A_{\infty }}=kt \end{array}$$

where *A*_0_, *A*_t_, and *A*_∞_ are the absorbance of the azobenzene chromophores at their corresponding wavelengths at times 0 and t and at the photo-stationary stage, respectively, and *k* is the rate constant of the photoisomerization process.

### Binding Kinetics of PS-*co*-PMAA@PSMIPs and PS-*co*-PMAA@PSNIPs for PFF

The adsorption kinetics of PS-*co*-PMAA@PSMIPs was studied. Approximately 5 mL PFF solution (300 μmol/L) in DMSO/H_2_O (3:1, v/v) and 20.0 mg PS-*co*-PMAA@PSMIPs were placed in each plastic centrifuge tube, sealed, and dispersed ultrasonically evenly. The obtained suspensions were incubated for different predetermined times (5, 15, 30, 45, 60, 90, 120, and 240 min, respectively) in the dark and then centrifuged at 10,000 rpm for 5 min. The supernatant was filtered through a 0.22-μm polyethersulfone syringe filter and then analyzed by UV-Vis spectrophotometry. The adsorption kinetics experiment of PS-*co*-PMAA@PSNIPs was measured using the same method. All experiments were repeated three times, and the averaged value was used. The binding capacity was calculated according to Equation 2 (Long et al., 2016),

(2) $$\begin{array}{r} Q\;\left({\text{mg g}}^{-1}\right)\;=\frac{\left(C_{0}-\;C_{s}\right)\;V}{m} \end{array}$$

where *C*_0_ and *C*_*s*_ represent the initial and equilibrium concentrations of PFF in solution (mg mL^−1^), *V* is the volume of the bulk solution (mL), and *m* is the mass (g) of the material.

### Adsorption Isotherms of PS-*co*-PMAA@PSMIPs/PS-*co*-PMAA@PSNIPs for PFF

Approximately 20.0 mg of PS-*co*-PMAA@PSMIPs or PS-*co*-PMAA@PSNIPs was suspended in 5-mL PFF solutions in DMSO/H_2_O (3:1, v/v) with various PFF concentrations, ultrasonically dispersed evenly, and sealed. The obtained suspension was incubated for 2 h in the dark and then centrifuged at 10,000 rpm for 5 min. The supernatant was filtered through a 0.22-μm polyethersulfone syringe filter and then measured by UV-Vis spectrophotometry. All experiments were repeated three times, and the averaged value was used. The adsorption kinetic capacity of PS-*co*-PMAA@PSMIPs for PFF (Q) was calculated according to Equation 3:

(3) $$\begin{array}{r} \frac{Q}{C_{s}}=\frac{Q_{\max}-Q}{K_{d}} \end{array}$$

where *Q*_*max*_, *Q, C*_*s*_, and *K*_*d*_ represent the maximal chemical binding capacity of PS-*co*-PMAA@PSMIPs for PFF (mg/g), the equilibrium adsorption capacity of PFF bound to polymers (mg/g), the equilibrium concentration of PFF in solution (mg/L), and the dissociation constant (mg/L).

The binding specificity of PS-*co*-PMAA@PSMIPs and PS-*co*-PMAA@PSNIPs was investigated by using the structural analogs CPF and CPFm as reference compounds. The specific research method is the same as described above, but 5 mL CPF or CPFm solution (300 μmol/L) in DMSO/H_2_O (3:1, v/v) was used.

### Photoregulated Release and Uptake Studies

For the purpose of studying the photoregulated release and uptake, 20.0 mg of PS-*co*-PMAA@PSMIPs or PS-*co*-PMAA@PSNIPs was suspended in 5 mL PFF solution (300 μmol/L) in DMSO/H_2_O (3:1, v/v). The obtained suspension was ultrasonically dispersed, sealed, and incubated for 2 h in the dark and centrifuged at 10,000 rpm for 5 min. The supernatant was filtered through a 0.22-μm polyethersulfone syringe filter and then measured by UV-Vis spectrophotometry. The adsorption capacity of PS-*co*-PMAA@PSMIPs or PS-*co*-PMAA@PSNIPs to PFF was calculated. PS-*co*-PMAA@PSMIPs or PS-*co*-PMAA@PSNIPs were re-dispersed in the supernatant to form a uniform suspension and then alternately irradiated at 365 nm for 100 min and 440 nm for 45 min. In the interval of alternating irradiation, the adsorption capacity of PS-*co*-PMAA@PSMIPs or PS-*co*-PMAA@PSNIPs to PFF was measured and calculated in the same way as described above.

### Determination of PFF in Tomato and Mangosteen

Approximately 20.00 g of the homogeneous tomato or mangosteen sample was added to two 50-mL plastic centrifuge tubes, to which 0.1 and 0.25 mg standard samples of PFF were, respectively, added. After incubating for 4 h, the spiked samples were extracted ultrasonically with 15 mL DMSO/H_2_O (3:1, v/v) solution for about 20 min. The above extraction was repeated two times. The mixture was centrifuged at 12,000 r/min for 5 min. The supernatant was subsequently filtered through a 0.22-μm polyethersulfone syringe filter, transferred into a 50-mL volumetric flask, and diluted to the mark with DMSO/H_2_O (3:1, v/v).

## Results and Discussion

### Synthesis of PS-*co*-PSMIPs and PS-*co*-PSNIPs

**Figure 1:**
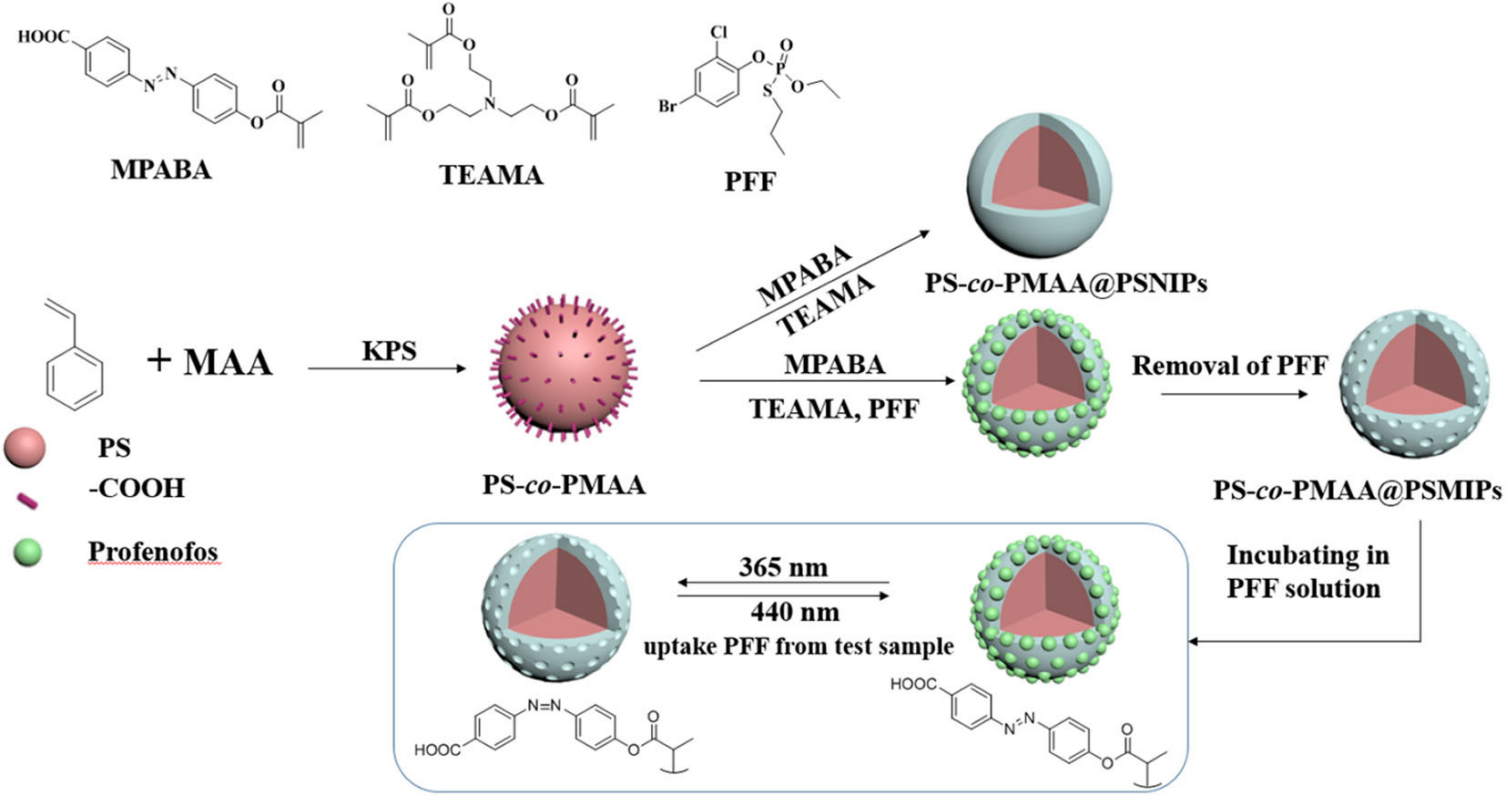
Synthetic procedure for PS-*co*-PMAA@PSMIPs and PS-*co*-PMAA@PSNIPs.

The preparation process of PS-*co*-PMAA@SMIPs is shown in Figure 1. A series of exploration experiments was conducted, and finally the optimum synthesis conditions were determined: PS-*co*-PMAA/MPABA = 5:3 (mass ratio) and MPABA/TEAMA = 1:2 (molar ratio) and using DMSO/H_2_O (3:1, v/v) as the solvent.

### Characterization of PS-*co*-PSMIPs and PS-*co*-PSNIPs

**Figure 2:**
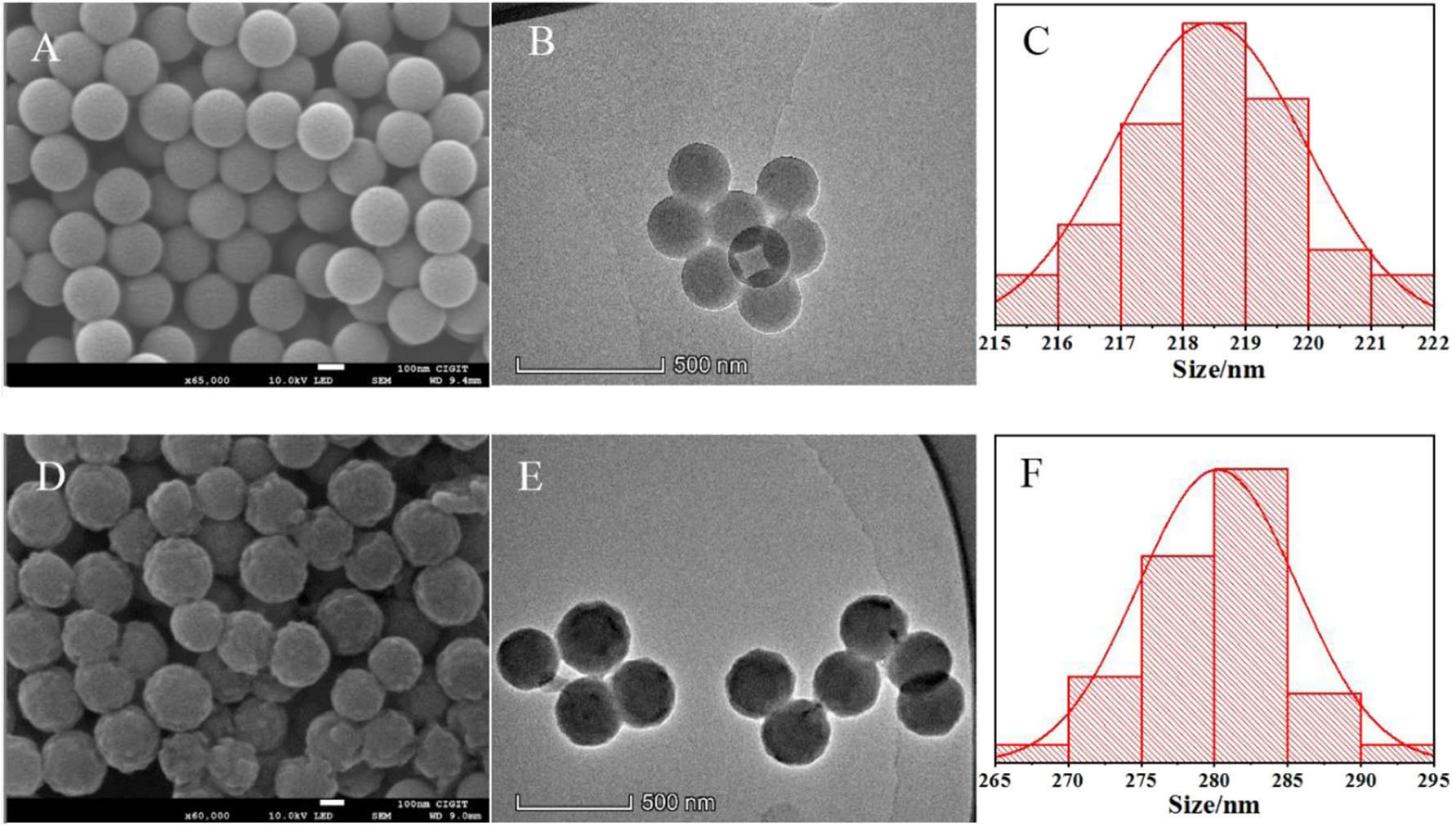
SEM microphotographs of PS-*co*-PMAA **(A)** and PS-*co*-PMAA@PSMIPs **(D)**. TEM microphotographs of PS-*co*-PMAA **(B)** and PS-*co*-PMAA@PSMIPs **(E)** and the normal size distribution of PS-*co*-PMAA **(C)** and PS-*co*-PMAA@PSMIPs **(F)**.

As shown in Figure 2, PS-*co*-PMAA with a smooth surface has a narrow distribution of 215–225 nm and the particle size is mainly about 218 nm in diameter, while the polymer particles PS-*co*-PMAA@PSMIPs and PS-*co*-PMAA@PSNIPs (Supplementary Figure 1) have a rough surface with a distribution of 265–295 nm and the particle size is mainly about 280 nm in diameter. This indicates that the copolymer layer of MPABA and TEAMA was uniformly grown on the substrate surface with a thickness of ~30 nm, since the carboxyl-modified polystyrene microspheres have good compatibility with the copolymer layer, which is conducive to the formation of a uniform shell layer on polystyrene matrices.

**Figure 3:**
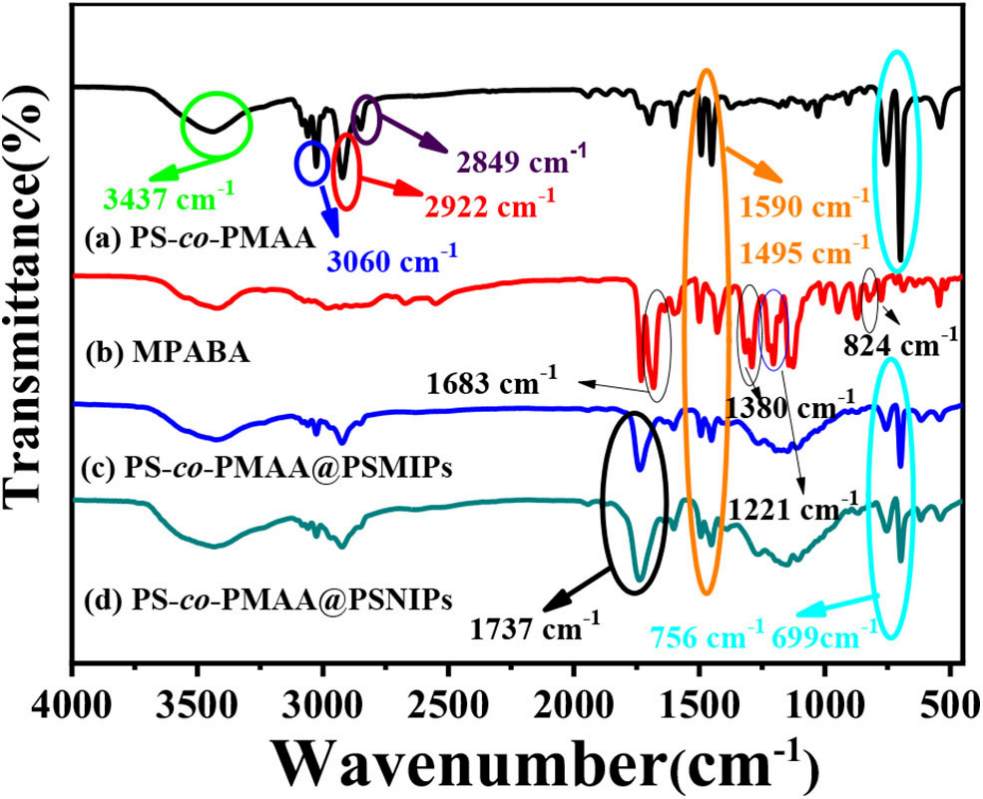
The infrared spectra of PS-*co*-PMAA **(a)**, MPABA **(b)**, PS-*co*-PMAA@PSMIPs **(c)**, and PS-*co*-PMAA@PSNIPs **(d)**.

As shown in Figure 3, in the polymer particles of PS-*co*-PMAA@PSMIPs and PS-*co*-PMAA@PSNIPs, the out-of-plane C–H bending vibration peaks of MPABA at 756 cm^−1^ and 699 cm^−1^ and the benzene-ring carbon skeleton stretching vibration peaks from PS-*co*-PMAA at 1,590 and 1,495 cm^−1^ are retained (Yang et al., 2018), and the stretching vibration peak at 1,737 cm^−1^ owing to C=O from cross-linkers is enhanced (Wang et al., 2019). This indicated that the monomer and the cross-linkers were successfully copolymerized on PS-co-PMAA.

**Figure 4:**
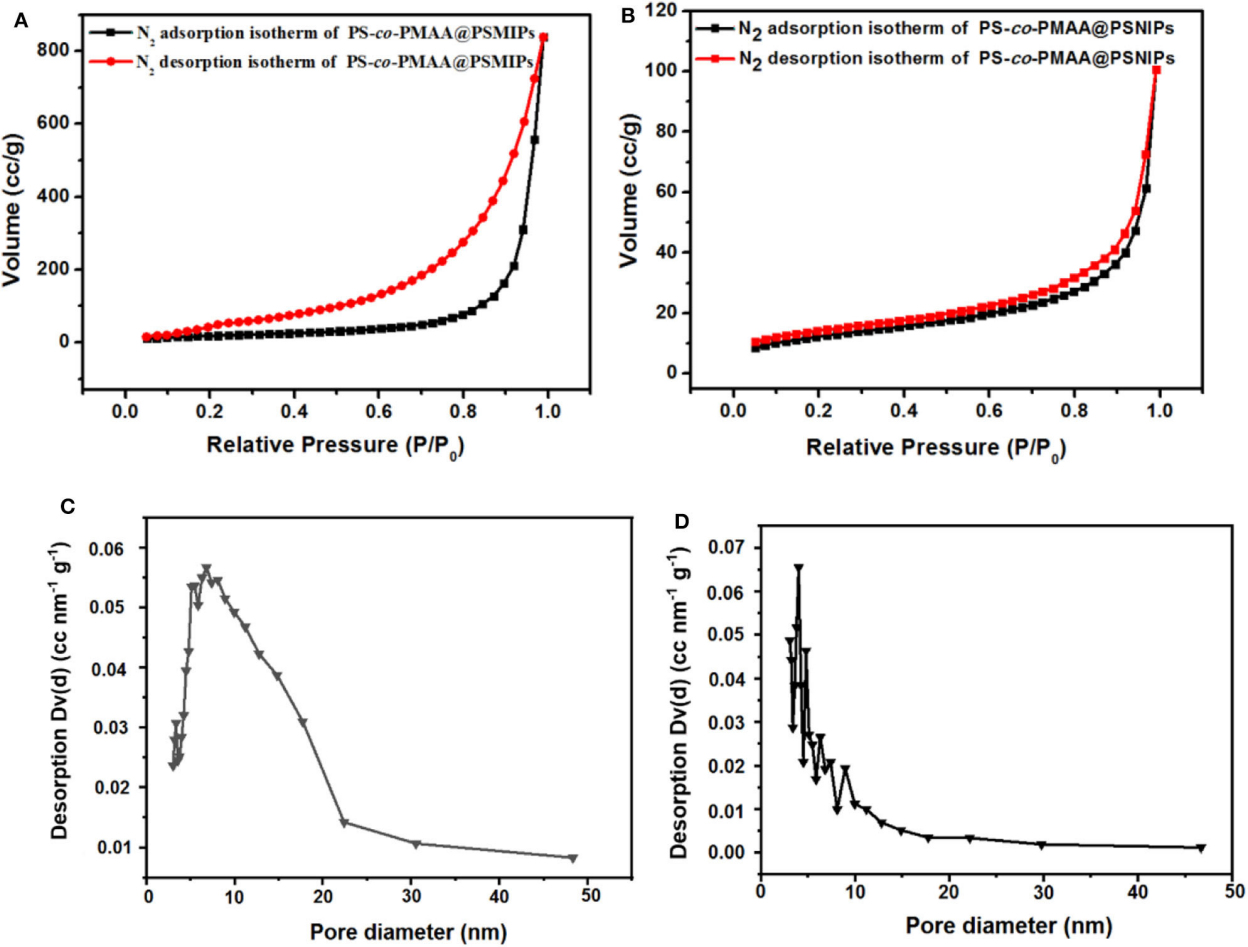
Nitrogen adsorption–desorption isotherms of PS-*co*-PMAA@PSMIPs **(A)** and PS-*co*-PMAA@PSMIPs **(B)** at 77 K. The pore size distribution of PS-*co*-PMAA@PSMIPs **(C)** and PS-*co*-PMAA@PSMIPs **(D)** based on the BJH method using the nitrogen adsorption data.

The porous nature of PS-*co*-PMAA@PSMIPs and PS-*co*-PMAA@PSNIPs was investigated by N_2_ adsorption–desorption analysis. As shown in Figure 4, the isotherms of PS-*co*-PMAA@PSMIPs and PS-*co*-PMAA@PSNIPs approached to type IV isotherms. The analysis results of the BET method for surface areas and the BJH method for pore volume are summarized in Table 1. PS-co-PMAA@PSMIPs had larger values in surface area, pore volume, and pore size owing to imprinted cavities.

**Table 1:** The surface area, pore diameter, and pore volume of PS-*co*-PMAA@PSMIPs and PS-*co*-PMAA@PSNIPs.

**Materials**	**Surface area (m^2^/g)**	**Pore volume (cc/g)**	**Pore size (nm)**
PS-*co*-PMAA@PSMIPs	72.6	1.21	6.8
PS-*co*-PMAA@PSNIPs	45.1	0.10	4.0

### Photoisomerization Analysis

**Figure 5:**
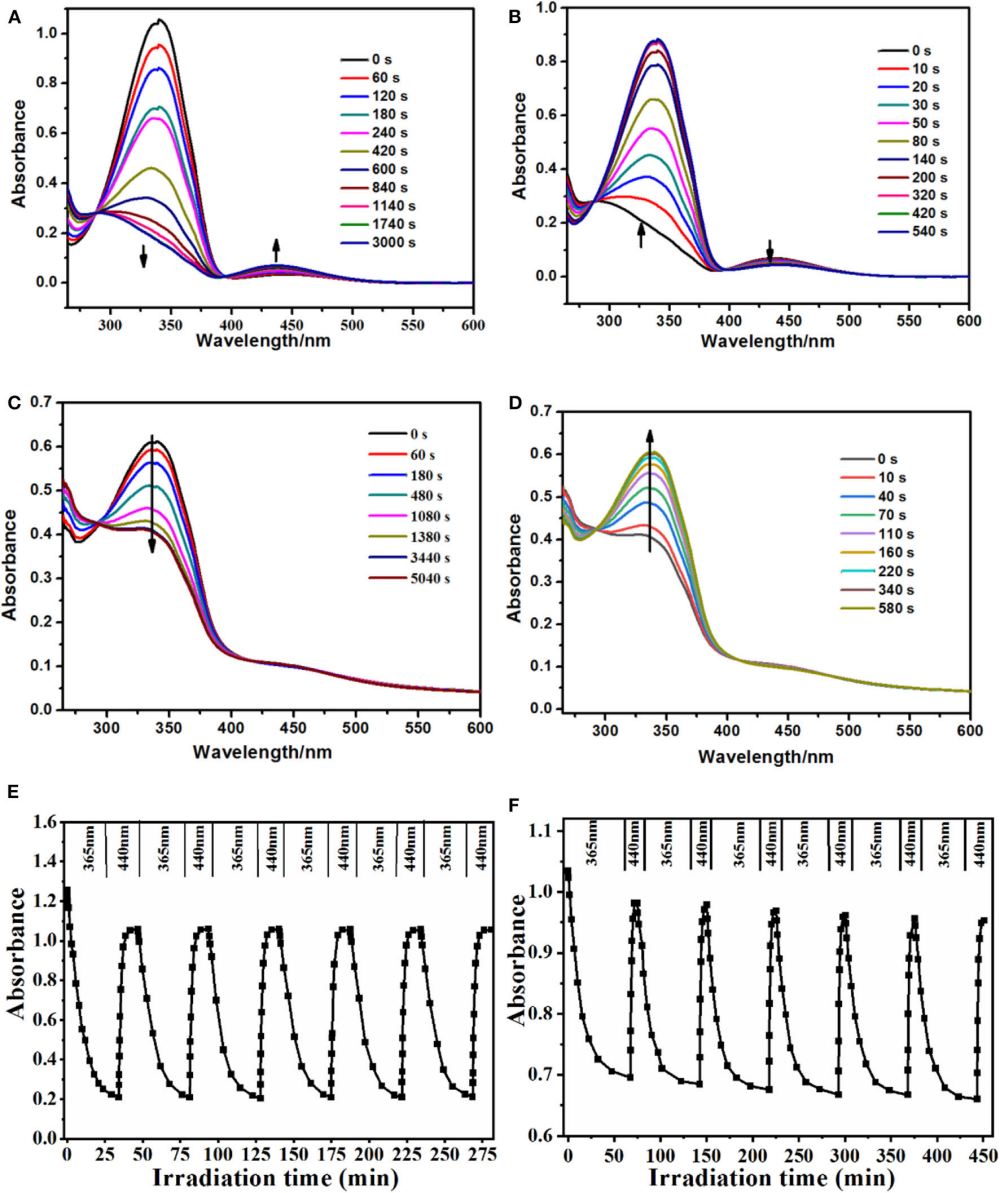
UV–vis spectra of MPABA (35 μmol/L) in DMSO/H_2_O (3:1, v/v) upon irradiation at 365 nm **(A)** and then at 440 nm **(B)**. UV–vis spectra of PS-*co*-PMAA@PSMIPs (0.2 mg/mL) in DMSO/H_2_O (3:1, v/v) upon irradiation at 365 nm **(C)** and then at 440 nm **(D)**. Reversibility of the photoisomerization processes of azobenzene chromophores in the MPABA **(E)** and PS-*co*-PMAA@PSMIPs **(F)** [0.2 mg/mL in DMSO/H_2_O (3:1, v/v)] upon alternate irradiation at 365 and 440 nm, respectively.

The spectroscopic responses of PS-*co*-PMAA@PSMIPs were consistent with MPABA (Figures 5A–D). This indicated that photoresponsive properties of azobenzene chromophores were successfully retained in PS-*co*-PMAA@PSMIPs, while the photoisomerization ability declined slightly since the rotation of azobenzene was prevented by a rigid three-dimensional imprinted copolymer layer (Table 2). The trans→ cis and cis→ trans photoisomerization rate constants of PS-*co*-PMAA@PSMIPs were 1.98- and 1.02-fold smaller than those of MPABA. The trend was consistent with previous reports (Gong et al., 2006, 2016; Li et al., 2015). Compared with the trans→ cis photoisomerization rate constant, the possible reason for the smaller decrease in cis→ trans photoisomerization rate constant was that sufficient free volume was created after trans→ cis photoisomerization. Nonetheless, under alternating irradiation at 365 and 440 nm, the photoisomerization reversibility of PS-*co*-PMAA@PSMIPs did not significantly decrease after five cycles (Figures 5E,F).

**Table 2:** Photoisomerization rate constant of MPABA and PS-*co*-PMAA@PSMIPs.

**Materials**	***k*_**(*trans*→ *cis*)**_ (s^**−1**^)**	***k*_**(*cis*→ *trans*)**_ (s^**−1**^)**
MPABA	(3.38 ± 0.08) × 10^−3^	(12.87 ± 0.20) × 10^−3^
PS-*co*-PMAA@PSMIPs	(1.71 ± 0.09) × 10^−3^	(12.67 ± 0.14) × 10^−3^

### Binding Kinetics

**Figure 6:**
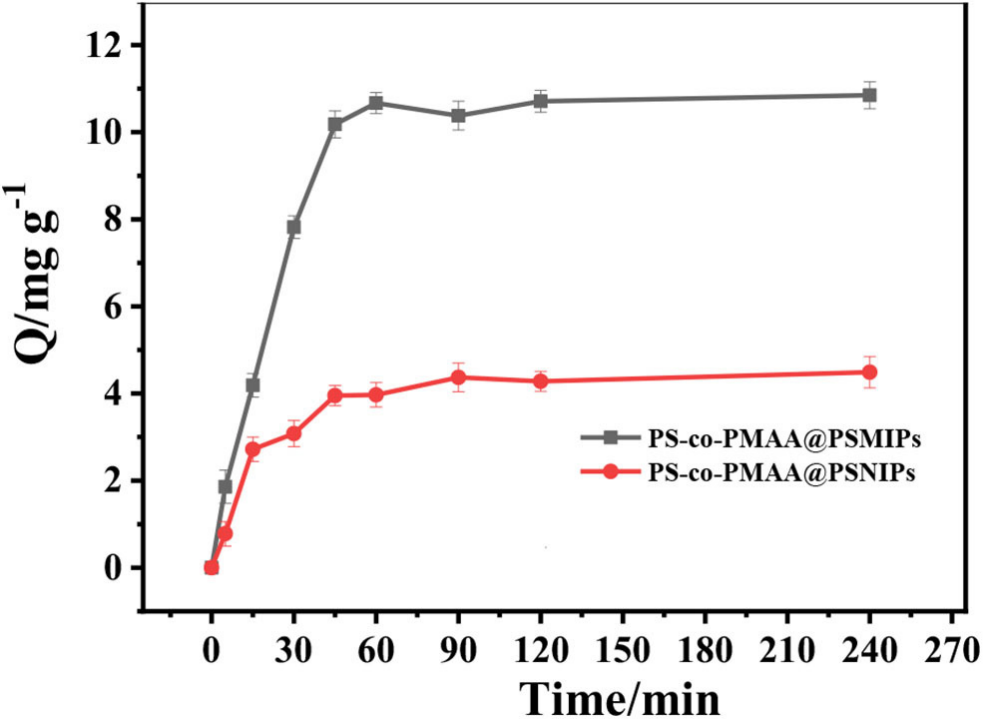
The relationship between binding capacity of PS-*co*-PMAA@PSMIPs/PS-*co*-PMAA@PSNIPs and binding time for PFF.

In DMSO/H_2_O (3:1, v/v), the adsorption intensity of PFF at 277 nm increased as its concentration increased (Supplementary Figure 3A), and a good linear relationship was observed between the absorbance at 277 nm and PFF concentration (Supplementary Figure 3B). Therefore, the absorbance at 277 nm was used to estimate the PFF concentration in the corresponding solution by using Supplementary Figure 3B as the standard curve. As shown in Supplementary Figure 4, the absorbance at 277 nm decreased as the incubation time increased; this illustrates that PFF in the solution was gradually absorbed by PS-*co*-PMAA@PSMIPs. An equilibrium was obtained after 60 min. Based on these data, the binding capacity of PS-*co*-PMAA@PSMIPs/PS-*co*-PMAA@VPSNIPs toward PFF was calculated using Equation 2. It is seen from Figure 6 and Supplementary Table 1 that the binding capacity of PS-*co*-PMAA@PSMIPs/PS-*co*-PMAA@VPSNIPs toward PFF shows a trend of increasing first within 60 min and then stabilizing. The binding capacity of PS-*co*-PMAA@PSMIPs (10.56 mg/g) was significantly higher than the PS-*co*-PMAA@VPSNIP material (4.38 mg/g) owing to the imprinted cavities. PS-*co*-PMAA@PSMIPs reached the adsorption equilibrium in about 45 min, while PS-*co*-PMAA@VPSNIPs took about 90 min. This was ascribed to the strong affinity of the imprinted cavities that made the template quickly bind to PS-*co*-PMAA@PSMIPs.

### Equilibrium Rebinding Study and Scatchard Analysis

**Figure 7:**
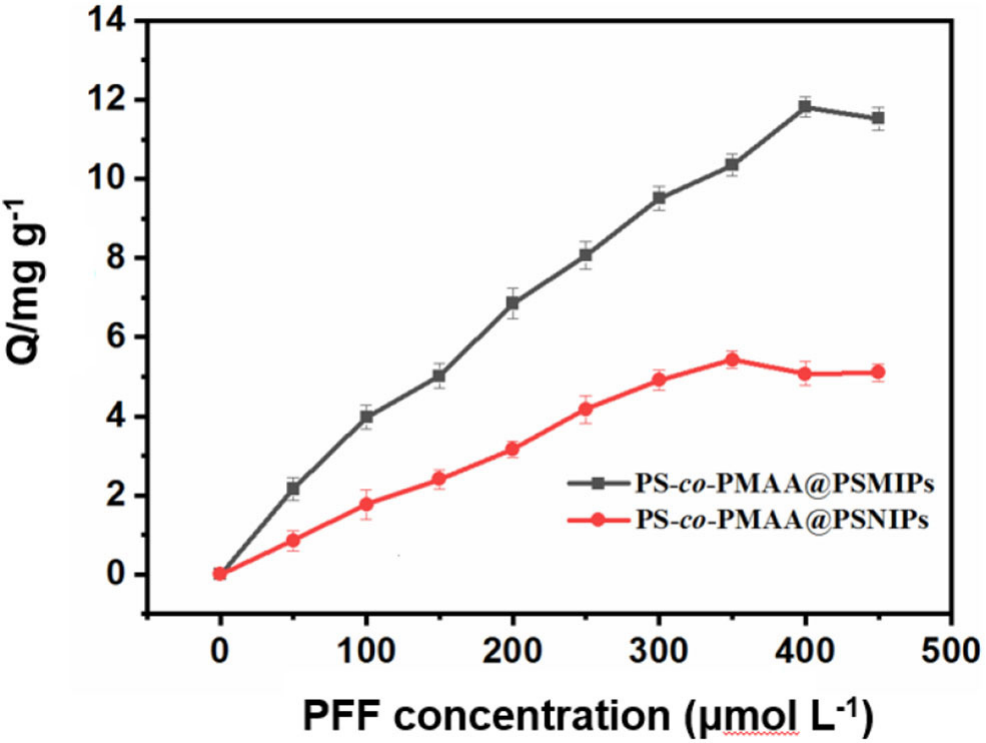
Static adsorption isotherms of PS-*co*-PMAA@PSMIPs/PS-*co*-PMAA@PSNIPs for profenofos (50~450 μmol/L).

As shown in the static isotherm of PS-*co*-PMAA@PSMIPs/PS-*co*-PMAA@PSNIPs to PFF (Figure 7), as the initial concentration of PFF increased, the binding capacity of PS-*co*-PMAA@PSMIPs/PS-*co*-PMAA@PSNIPs increased rapidly, then increased slowly, and finally reached a plateau. PS-*co*-PMAA@PSMIPs exhibited a faster binding rate than PS-co-PMAA@PSNIPs. The maximum binding capacity of PS-*co*-PMAA@PSMIPs toward PFF for was 11.82 mg/g, which was about twice of PS-*co*-PMAA@PSNIPs (5.43 mg/g).

**Figure 8:**
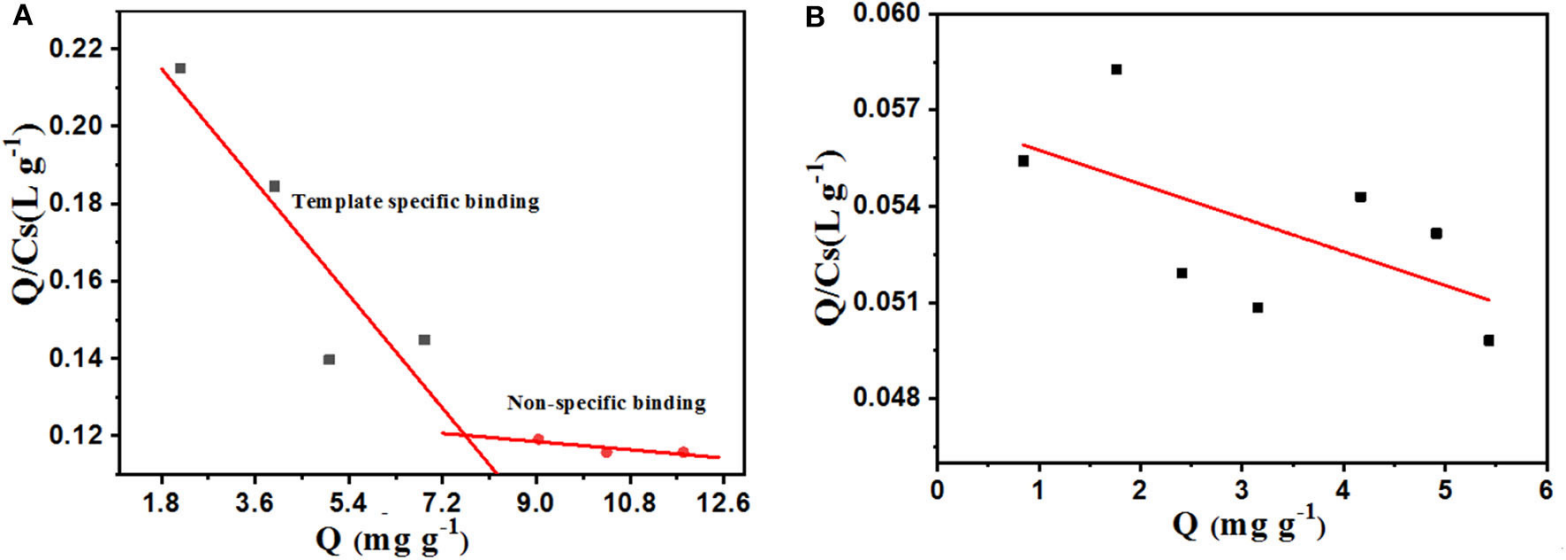
Scatchard plot for profenofos in PS-*co*-PMAA@PSMIPs **(A)** and PS-*co*-PMAA@PSNIPs **(B)**.

As shown in Figure 8, the isothermal adsorption data of PS-*co*-PMAA@PSMIPs was analyzed by the Scatchard model. Two straight lines with different slopes were observed, which indicated that there were two types of binding sites on PS-*co*-PMAA@PSMIPs for PFF, namely, high-affinity sites and low-affinity sites. The steeper straight line originated from the strong affinity of the imprinted cavities to PFF, from which *K*_d_ was calculated as 61.35 mg/L, and *Q*_max_ was 14.98 mg/g, while the flatter line showed the weak affinity binding capacity owing to physical adsorption on the surface of PS-*co*-PMAA@PSMIPs. PS-*co*-PMAA@PSNIPs had only a weak physical binding property due to the absence of the imprinted cavities of PFF.

### Binding Selectivity

**Figure 9:**
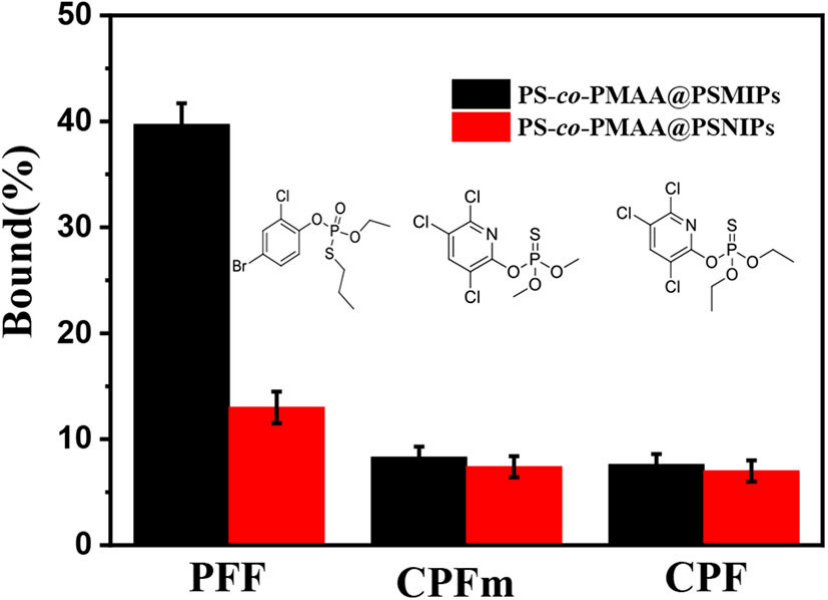
Selective adsorption capacity of PS-*co*-PMAA@PSMIPs/PS-*co*-PMAA@PSNIPs for PFF, CPFm, and CPF.

The binding capacity of PS-*co*-PMAA@PSMIPs to PFF, CPFm, and CPF was, respectively about 39.7, 8.3, and 7.6% (Figure 9). The binding capacities of PS-*co*-PMAA@PSMIPs to PFF were, respectively, 4.8- and 5.2-fold larger than those of PFF CPFm and CPF. The difference in binding capacity came from the matching degree of the imprinted cavities, shape, and size to the binding molecule. The above results indicated that PS-*co*-PMAA@PSMIPs had a specific binding capacity to PFF, while the binding capacity of PS-*co*-PMAA@PSNIPs to PFF, CPFm, and CPF was, respectively about 13.0, 7.4, and 7.0%. This demonstrated that PS-*co*-PMAA@PSNIPs only had nonspecific binding capacity to PFF.

### Photoregulated Release and Uptake of PFF

**Figure 10:**
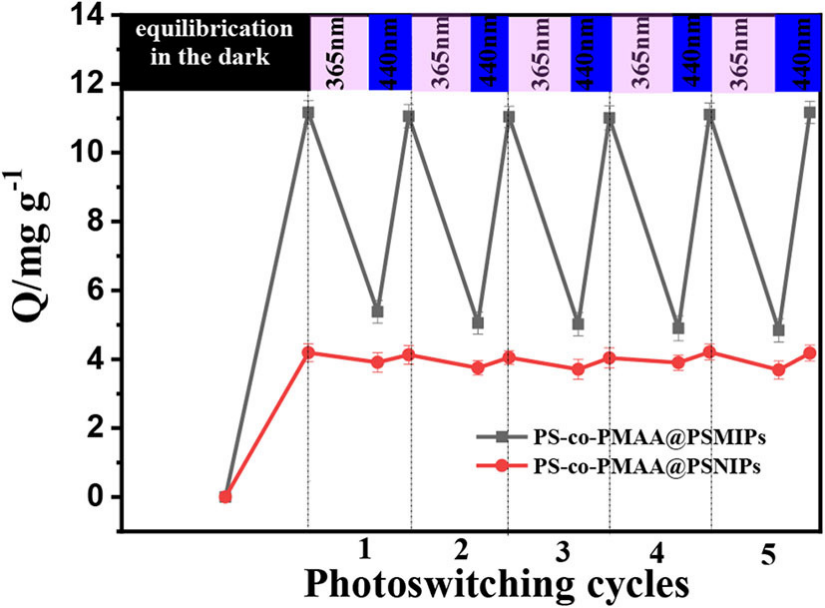
Photoregulated release and uptake of PFF by PS-*co*-PMAA@PSMIPs and PS-*co*-PMAA@PSNIPs.

The photoregulated release and uptake of PS-*co*-PMAA@PSMIPs for PFF were investigated under alternating irradiation at 365 and 440 nm in DMSO/H_2_O (3:1, v/v) solution. The process was repeated five times. As shown in Figure 10, when the material reached the adsorption equilibrium in the dark, imprinting cavities on PS-*co*-PMAA@PSMIPs were fully filled with PFF. Under irradiation at 365 nm, the azobenzene chromophore in PS-*co*-PMAA@PSMIPs underwent a configuration change from trans to cis. During this process, the shape and size of the PFF-imprinted cavities changed; this caused the PFF bound in the imprinted cavities to be squeezed or loosened and then fall from the cavities into the solution. Therefore, the binding capacity of PS-*co*-PMAA@PSMIPs for PFF was reduced from 11.06 mg/g to 5.05 mg/g. Upon irradiation at 440 nm, the azobenzene chromophore in PS-*co*-PMAA@PSMIPs underwent a configuration change from cis to trans, which caused the PFF-imprinted cavities to return to its original shape and size. During this process, PFF was rebound in the imprinted cavities due to the affinity of the cavities. So the binding capacity of PS-*co*-PMAA@PSMIPs for PFF was increased from 5.05 to 11.04 mg/g. During a repeating in the photo-switching cycle, the release and uptake of PFF were similar to the previous cycle. This excellent capacity, however, was not working on PS-*co*-PMAA@PSNIPs because of its non-imprinted cavities.

### Determination of Trace PFF in Spiked Mangosteen and Tomato Samples

**Figure 11:**
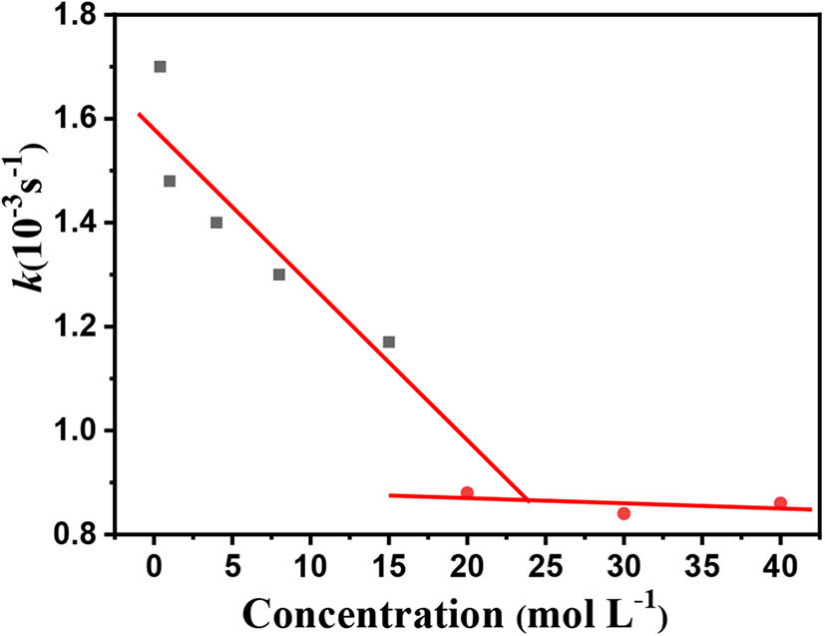
Photoisomerization rate constant (trans→ cis) of the PS-*co*-PMAA@PSMIPs vs. PFF concentration.

As shown in Figure 11, the imprinted cavities inside the PS-*co*-PMAA@PSMIPs were gradually filled as the PFF concentration increased, and the photoisomerization rate constant (*k*) of PS-*co*-PMAA@PSMIPs decreased firstly. As the PFF concentration increased to 20 μmol/L, *k* tended to be invariant. When the concentration of PFF reached a certain level, the imprinted cavities of PS-*co*-PMAA@PSMIPs were filled with PFF. There was insufficient free volume for azobenzene chromophore of the imprinted materials to rotate, thus making isomerization more difficult. *k* showed a good linear relationship with the PFF concentration in the range of 0~15 μmol/L. The detection limit was 0.40 μmol/L (0.35 mg/kg), which is below the maximum residue limit (10 mg/kg) for PFF in mangosteen and tomato samples stipulated by the National Standards of the People's Republic of China (GB-2763-2019). The feasibility of the test method was evaluated by detecting trace PFF in mangosteen or tomato samples at two spiked concentrations (2.00 mg/L, 5.00 mg/L). As shown in Table 3, the recovery data for spiked PFF standards ranged from 94.4 to 102.4 and relative standard deviation (RSD%) ranged from 2.4 to 5.9%. Therefore, the concentration of PFF in the detection system can be calculated by measuring the photoisomerization rate constant of PS-*co*-PMAA@PSMIPs. Hence, a convenient method for determining trace PFF by ultraviolet light response was established.

**Table 3:** Detection of PFF in the spiked real samples at different concentrations (*n* = 3).

**Samples**	**Determined**	**Spiked (mg/L)**	**Measured (mg/L)**	**Recovery (%)**	**RSD (%)**
Tomato	Not detected	2.00	2.05	102.4	5.9
	Not detected	5.00	4.94	98.8	2.4
Mangosteen	Not detected	2.00	1.89	94.4	7.4
	Not detected	5.00	4.86	99.6	3.7

In comparison with reported methods for detecting PFF (Raharjo et al., 2009; Yang et al., 2012; Zhang et al., 2014; Li C. et al., 2018), the method in this work had a higher detection limit (Table 4). However, this value (0.35 mg/kg) is below the maximum residue limit (10 mg/kg) for PFF in mangosteen and tomato samples stipulated by the National Standards of the People's Republic of China (GB-2763-2019) and can satisfy the requirement in this field. It is noteworthy that the sample preparation is simple, and expensive instruments or special materials (e.g., biomaterials) are required.

**Table 4:** Comparison of reported methods for detection of PFF with this work.

**Detection method**	**Instrument**	**Sample preparation**	**Detection limit (mg/kg)**	**References**
PS-*co*-PMAA@PSMIP-based method	UV-Vis spectrophotometry	Simple	0.35	This work
Gas chromatography	Gas chromatography	Tedious	0.001–0.025	Yang et al., 2012
HPLC	HPLC	Complicated	0.018	Raharjo et al., 2009
Biosensor	Microcantilever-array instrument	Simple	0.003	Li C. et al., 2018
Fluorescent sensor	Mithras LB 940 multimode microplate reader	Biomaterial required	0.012	Zhang et al., 2014

## Conclusion

In summary, photoresponsive surface molecularly imprinted polymers (PS-*co*-PMAA@PSMIPs) with a uniform shell thickness were prepared by combining stimulus-responsive polymers with surface molecular imprinting technology. The hydrogen bond driving effect from the PS-*co*-PMAA surface and MPABA and the good compatibility between the PS-*co*-PMAA-based organic substrate and the copolymer layer are all conducive to the formation of a homogeneous shell polymer, which contributed to the binding capacity of PS-*co*-PMAA@PSMIPs. The photoresponsive properties of azobenzene chromophore were well-retained. PS-*co*-PMAA@PSMIPs can specifically bind PFF. PS-co-PMAA@PSMIPs were applied to detect trace PFF in fruit and vegetable samples with complex matrices with good recoveries and low relative standard deviation.

## Data Availability Statement

All datasets generated for this study are included in the article/Supplementary Material.

## Author Contributions

MC performed the experiments and wrote the draft. HY and YS performed parts of the experiments. QT provided some ideas, discussed, and revised the manuscript. CC provided some ideas and discussed. CG designed, supervised, and revised the manuscript. All authors contributed to the article and approved the submitted version.

## Conflict of Interest

The authors declare that the research was conducted in the absence of any commercial or financial relationships that could be construed as a potential conflict of interest.

## References

[B1] Alaei H. S. Tehrani M. S. Husain S. W. Panahi H. A. Mehramizi A. (2018). Photo-regulated ultraselective extraction of Azatioprine using a novel photoresponsive molecularly imprinted polymer conjugated hyperbranched polymers based magnetic nano-particles. Polymer 148, 191–201. 10.1016/j.polymer.2018.06.013

[B2] Altintas Z. Abdin M. J. Tothill A. M. Karim K. Tothill I. E. (2016). Ultrasensitive detection of endotoxins using computationally designed nanoMIPs. Anal. Chim. Acta 935, 239–248. 10.1016/j.aca.2016.06.013 27543033

[B3] Boitard C. Curcio A. Rollet A. L. Wilhelm C. Menager C. Griffete N. (2019). Biological fate of magnetic protein-specific molecularly imprinted polymers: toxicity and degradation. ACS Appl. Mater. Interfaces 11, 35556–35565. 10.1021/acsami.9b11717 31496222

[B4] Dadson O. A. Ellison C. A. Singleton S. T. Chi L. H. Mcgarrigle B. P. Lein P. J.. (2013). Metabolism of profenofos to 4-bromo-2-chlorophenol, a specific and sensitive exposure biomarker. Toxicology 306, 35–39. 10.1016/j.tox.2013.01.023 23415833 PMC4751995

[B5] Ding S. Lyu Z. Niu X. Zhou Y. Liu D. Falahati M.. (2020). Integrating ionic liquids with molecular imprinting technology for biorecognition and biosensing: a review. Biosens. Bioelectron. 149:111830. 10.1016/j.bios.2019.111830 31710919

[B6] Gomez-Arribas L. N. Urraca J. L. Benito-Pena E. Moreno-Bondi M. C. (2019). Tag-specific affinity purification of recombinant proteins by using molecularly imprinted polymers. Anal. Chem. 91, 4100–4106. 10.1021/acs.analchem.8b05731 30786715

[B7] Gong C. B. Lam M. H. W. Yu H. X. (2006). The fabrication of a photoresponsive molecularly imprinted polymer for the photoregulated uptake and release of caffeine. Adv. Funct. Mater. 16, 1759–1767. 10.1002/adfm.200500907

[B8] Gong C. B. Li Z. Y. Liu L. T. Wei Y. B. Yang X. Chow C. F. (2017a). Photocontrolled extraction of uric acid from biological samples based on photoresponsive surface molecularly imprinted polymer microspheres. J. Sep. Sci. 40, 1396–1402. 10.1002/jssc.201601243 28106341

[B9] Gong C. B. Ou X. X. Liu S. Jin Y. L. Huang H. R. Tang Q. (2017b). A molecular imprinting-based multifunctional chemosensor for phthalate esters. Dyes Pigments 137, 499–506. 10.1016/j.dyepig.2016.10.047

[B10] Gong C. B. Wei Y. B. Liu L. T. Zheng A. X. Yang Y. H. Chow C. F.. (2017c). Photoresponsive hollow molecularly imprinted polymer for trace triamterene in biological samples. Mater. Sci. Eng. C 76, 568–578. 10.1016/j.msec.2017.03.135 28482565

[B11] Gong C. B. Yang Y. H. Chen M. J. Liu L. T. Liu S. Wei Y. B. (2019). A photoresponsive molecularly imprinted polymer with rapid visible-light-induced photoswitching for 4-ethylphenol in red wine. Mater. Sci. Eng. C 96, 661–668. 10.1016/j.msec.2018.11.089 30606579

[B12] Gong C. B. Yang Y. Z. Yang Y. H. Zheng A. X. Liu S. Tang Q. (2016). Photoresponsive hollow molecularly imprinted polymer for the determination of trace bisphenol A in water. J. Colloid Interface. Sci. 481, 236–244. 10.1016/j.jcis.2016.07.039 27478978

[B13] Gotoh M. Sakata M. Endo T. Hayashi H. Seno H. Suzuki O. (2001). Profenofos metabolites in human poisoning. Forensic Sci Int. 116, 221–226. 10.1016/S0379-0738(00)00377-7 11182275

[B14] He J. Fan M. T. Liu X. J. (2010). Environmental behavior of profenofos under paddy field conditions. Bull Environ. Contam. Toxicol. 84, 771–774. 10.1007/s00128-010-0023-z 20437027

[B15] He X. Lian Z. Wang J. (2018). Selective separation and purification of beta-estradiol from marine sediment using an optimized core-shell molecularly imprinted polymer. J. Sep. Sci. 41, 3848–3854. 10.1002/jssc.201800722 30152918

[B16] Kalecki J. Cieplak M. Dabrowski M. Lisowski W. Kuhn A. Sharma P. S. (2020). Hexagonally packed macroporous molecularly imprinted polymers for chemosensing of follicle-stimulating hormone protein. ACS Sens. 5, 118–126. 10.1021/acssensors.9b01878 31845570

[B17] Kovida S. V. Koner A. L. (2020). Rapid on-site and naked-eye detection of common nitro pesticides with ionic liquids. Analyst 145, 4335–4340. 10.1039/D0AN00452A 32377662

[B18] Li C. Zhang G. Wu S. Zhang Q. (2018). Aptamer-based microcantilever-array biosensor for profenofos detection. Anal. Chim. Acta 1020, 116–122. 10.1016/j.aca.2018.02.072 29655422

[B19] Li S. Pang C. Ma X. Zhao M. Li H. Wang M.. (2020). Chiral drug fluorometry based on a calix[6]arene/molecularly imprinted polymer double recognition element grafted on nano-C-dots/Ir/Au. Microchim. Acta 187:394. 10.1007/s00604-020-04356-x 32556561

[B20] Li X. Cui H. Zeng Z. (2018). A simple colorimetric and fluorescent sensor to detect organophosphate pesticides based on adenosine triphosphate-modified gold nanoparticles. Sensors 18:4302. 10.3390/s18124302 30563245 PMC6308458

[B21] Li Z. Y. Quan H. J. Gong C. B. Yang Y. Z. Tang Q. Wei Y. B.. (2015). Photocontrolled solid-phase extraction of guanine from complex samples using a novel photoresponsive molecularly imprinted polymer. Food Chem. 172, 56–62. 10.1016/j.foodchem.2014.09.027 25442523

[B22] Liu H. D. Zheng A. X. Gong C. B. Ma X. B. Lam M. H. W. Chow C. F. (2015). A photoswitchable organocatalyst based on a catalyst-imprinted polymer containing azobenzene. RSC Adv. 5, 2539–2542. 10.1039/C5RA10343F

[B23] Liu L. T. Chen M. J. Yang H. L. Huang Z. J. Tang Q. Chow C. F.. (2020). An NIR-light-responsive surface molecularly imprinted polymer for photoregulated drug release in aqueous solution through porcine tissue. Mater. Sci. Eng. C 106:110253. 10.1016/j.msec.2019.110253 31753332

[B24] Liu L. T. Li N. Chen M. J. Yang H. L. Tang Q. Gong C. B. (2018). Visible-light-responsive surface molecularly imprinted polymer for acyclovir through chicken skin tissue. ACS Appl. Bio Mater. 1, 845–852. 10.1021/acsabm.8b00275 34996176

[B25] Long Z. Xu W. Lu Y. Qiu H. (2016). Nanosilica-based molecularly imprinted polymer nanoshell for specific recognition and determination of rhodamine B in red wine and beverages. J. Chromatogr. B Anal. Technol. Biomed. Life Sci. 1029–1030, 230–238. 10.1016/j.jchromb.2016.06.030 27372912

[B26] Ma M. B. Dong S. Z. Jin W. H. Zhang C. Q. Zhou W. L. (2019). Fate of the organophosphorus pesticide profenofos in cotton fiber. J. Environ. Sci. Health B 54, 70–75. 10.1080/03601234.2018.1505036 30633718

[B27] Muratsugu S. Shirai S. Tada M. (2020). Recent progress in molecularly imprinted approach for catalysis. Tetrahedron Lett. 61:151603 10.1016/j.tetlet.2020.151603

[B28] Raharjo Y. Sanagi M. M. Ibrahim W. A. Naim A. A. Aboul-Enein H. Y. (2009). Application of continual injection liquid-phase microextraction method coupled with liquid chromatography to the analysis of organophosphorus pesticides. J. Sep. Sci. 32, 623–629. 10.1002/jssc.200800566 19165835

[B29] Rutkowska M. Płotka-Wasylka J. Morrison C. Wieczorek P. P. Namieśnik J. Marć M. (2018). Application of molecularly imprinted polymers in analytical chiral separations and analysis. Trends Anal. Chem. 102, 91–102. 10.1016/j.trac.2018.01.011

[B30] Schumers J. M. Fustin C. A. Gohy J. F. (2010). Light-responsive block copolymers. Macromol. Rapid Commun. 31, 1588–1607. 10.1002/marc.201000108 21567570

[B31] Selvolini G. Bajan I. Hosu O. Cristea C. Sandulescu R. Marrazza G. (2018). DNA-based sensor for the detection of an organophosphorus pesticide: profenofos. Sensors-Basel 18:2035. 10.3390/s18072035 29941847 PMC6068880

[B32] Tang Q. Li Z. Y. Wei Y. B. Yang X. Liu L. T. Gong C. B. (2016). Photoresponsive surface molecularly imprinted polymer on ZnO nanorods for uric acid detection in physiological fluids. Mater. Sci. Eng. C 66, 33–39. 10.1016/j.msec.2016.03.082 27207036

[B33] Tang Q. Nie Y. T. Gong C. B. Chow C. F. Peng J. D. Lam M. H. W. (2012). Photo-responsive molecularly imprinted hydrogels for the detection of melamine in aqueous media. J. Mater. Chem. 22, 19812–19820. 10.1039/c2jm34522f

[B34] Wang Z. Qiu T. Guo L. Ye J. He L. Li X. (2019). The building of molecularly imprinted single hole hollow particles: a miniemulsion polymerization approach. Chem. Eng. J. 357, 348–357. 10.1016/j.cej.2018.09.128

[B35] Xiong S. Deng Y. Zhou Y. Gong D. Xu Y. Yang L. (2018). Current progress in biosensors for organophosphorus pesticides based on enzyme functionalized nanostructures: a review. Anal. Methods 10, 5468–5479. 10.1039/C8AY01851K

[B36] Xu S. Lu H. Zheng X. Chen L. (2013). Stimuli-responsive molecularly imprinted polymers: versatile functional materials. J. Mater. Chem. C 1, 4406–4422. 10.1039/c3tc30496e

[B37] Yang L. Li H. Zeng F. Liu Y. Li R. Chen H.. (2012). Determination of 49 organophosphorus pesticide residues and their metabolites in fish, egg, and milk by dual gas chromatography-dual pulse flame photometric detection with gel permeation chromatography cleanup. J. Agric. Food Chem. 60, 1906–1913. 10.1021/jf2043828 22300587

[B38] Yang Y. H. Liu L. T. Chen M. J. Liu S. Gong C. B. Wei Y. B.. (2018). A photoresponsive surface molecularly imprinted polymer shell for determination of trace griseofulvin in milk. Mater. Sci. Eng. C 92, 365–373. 10.1016/j.msec.2018.06.069 30184762

[B39] Zhang C. Wang L. Tu Z. Sun X. He Q. Lei Z.. (2014). Organophosphorus pesticides detection using broad-specific single-stranded DNA based fluorescence polarization aptamer assay. Biosens. Bioelectron. 55, 216–219. 10.1016/j.bios.2013.12.020 24384262

[B40] Zhang Y. Tan X. Liu X. Li C. Zeng S. Wang H. (2018). Fabrication of multilayered molecularly imprinted membrane for selective recognition and separation of artemisinin. ACS Sustainable Chem. Eng. 7, 3127–3137. 10.1021/acssuschemeng.8b04908

[B41] Zhao Q. Yang J. Zhang J. Wu D. Tao Y. Kong Y. (2019). Single-template molecularly imprinted chiral sensor for simultaneous recognition of alanine and tyrosine enantiomers. Anal. Chem. 91, 12546–12552. 10.1021/acs.analchem.9b03426 31476861

[B42] Zheng A. X. Gong C. B. Zhang W. J. Tang Q. Huang H. R. Chow C. F. (2017). An amphiphilic and photoswitchable organocatalyst for the aldol reaction based on a product-imprinted polymer. Mol. Catal. 442, 115–125. 10.1016/j.mcat.2017.07.022

