# Supplementary material for: Photoresponsive Surface Molecularly Imprinted Polymers for the Detection of Profenofos in Tomato and Mangosteen
Source: Front Chem. 2020 Oct 9;8:583036. doi: 10.3389/fchem.2020.583036 (PMC7581910; doi:10.3389/fchem.2020.583036)
Supplement: Supplementary file 1 [file Data_Sheet_1.PDF]

Supplementary Material

1. Morphology of PS-*co*-PMAA@PSNIPs

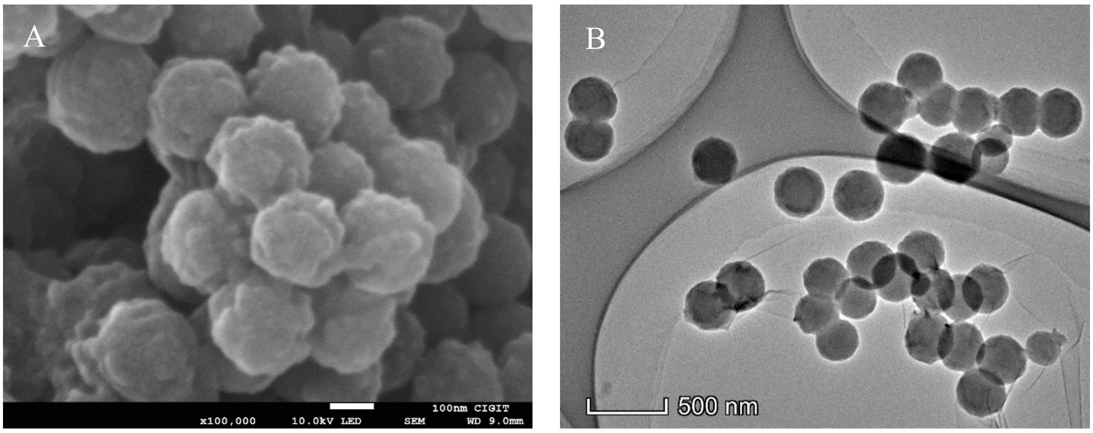

FIGURE S1 SEM (A) and TEM (B) microphotographs of PS-*co*-PSNIPs.

2. Kinetics of the trans→cis and cis→trans photoisomerizations

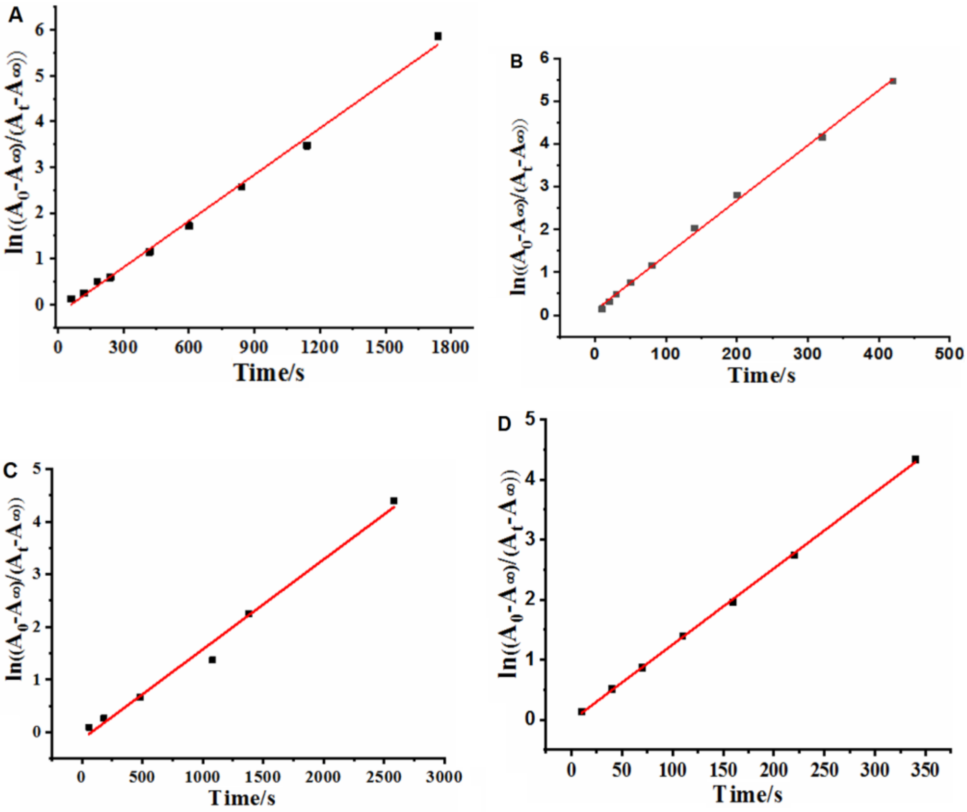

FIGURE S2 Kinetics of the trans→cis and cis→trans photoisomerizations of MPABA (A and B) and PS-*co*-PMAA@PSMIPs.

3. UV-spectra of PFF at different concentrations

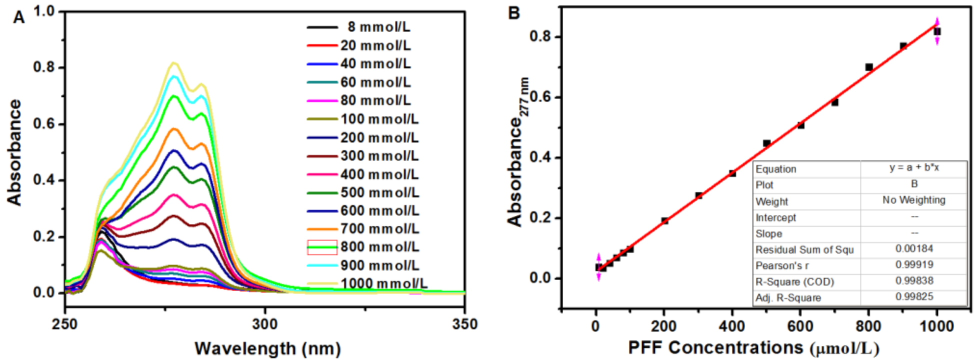

FIGURE S3 UV-Vis spectra of PFF in DMSO/H_2O (3:1, v/v) at different concentrations (A) and the relationship between the absorbance at 277 nm and PFF concentrations (B).

4. Binding kinetics of PS-*co*-PMAA@PSMIPs and PS-co-PMAA@PSNIPs to PFF

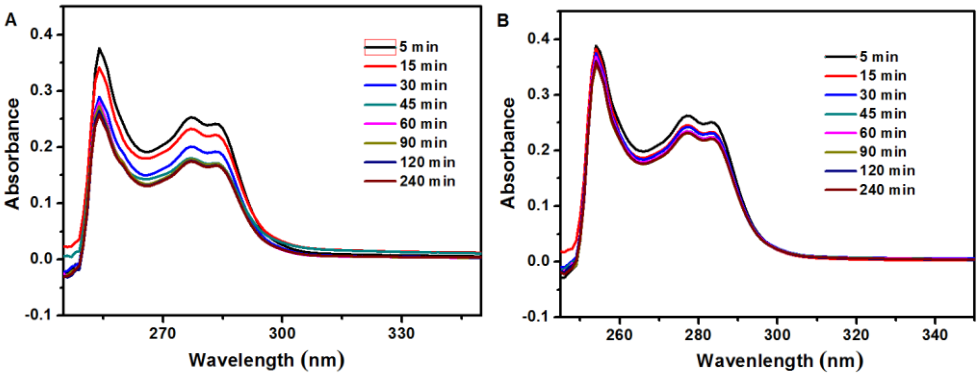

FIGURE S4 UV−Vis spectra of PFF (300 μmol/L) in DMSO/H_2O (3:1, v/v) upon the binding of PS-co-PMAA@PSMIPs (A) and PS-co-PMAA@PSNIPs (B) with different time

Table S1 The binding capacity of PS-co-PMAA@PSMIPs and PS-co-PMAA@PSNIPs

	PS-co-PMAA@PSMIPs					PS-co-PMAA@PSNIPs		
**t/min**	45	60	90	120	240	90	120	240
**Q_t /mg g^{-1}**	10.18	10.67	10.38	10.71	10.85	4.37	4.28	4.49
**Q/mg g^{-1}**			10.56				4.38	

Q_t represents the amount of binding capacity at the corresponding time; Q represents the average binding capacity after reaching equilibrium

5. Stability of PFF against ultraviolet irradiation

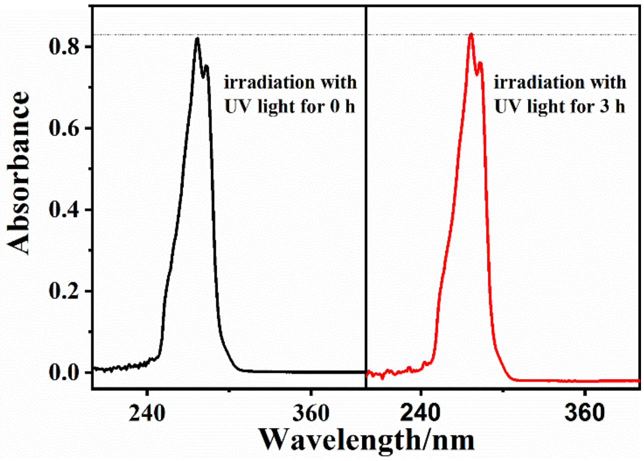

FIGURE S5 UV−Vis spectra of PFF (1mmol/L) in DMSO/H_2O (3:1, v/v) upon irradiation at 365 nm for 0 h (Black curve) and 3h (red curve).

6. Photoregulated uptake and release of PFF

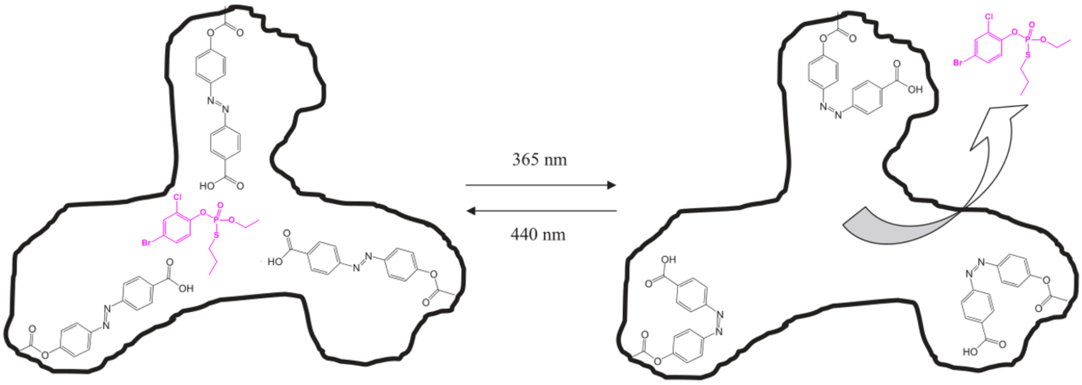

FIGURE S5 Schematic diagram for the photoregulated uptake and release of PFF
